# Percutaneous Electrolysis for Musculoskeletal Disorders Management in Rehabilitation Settings: A Systematic Review

**DOI:** 10.3390/healthcare13151793

**Published:** 2025-07-23

**Authors:** Carmelo Pirri, Nicola Manocchio, Andrea Sorbino, Nina Pirri, Calogero Foti

**Affiliations:** 1 Department of Neurosciences, Institute of Human Anatomy, University of Padua, 35121 Padova, Italy; 2 Physical and Rehabilitation Medicine, Department of Clinical Sciences and Translational Medicine, University of Rome “Tor Vergata”, 00133 Rome, Italy; andrea.sorbino@ptvonline.it (A.S.); foti@med.uniroma2.it (C.F.); 3 Department of Medicine—DIMED, School of Radiology, Radiology Institute, University of Padova, 35121 Padova, Italy; nina_92_@hotmail.it

**Keywords:** Percutaneous Electrolysis Therapy (EPTE), Electrolysis Percutaneous Intratissue (EPI), physiatry, electroacupuncture, elbow Tendinopathy, plantar fasciitis, patellar Tendinopathy, plantar heel pain, muscle injury

## Abstract

**Background**: Percutaneous electrolysis (PE) is a minimally invasive procedure that utilizes galvanic current delivered through a needle. PE is increasingly employed for musculoskeletal disorders, despite the scarcity of scientific evidence supporting its use. The aim of this systematic review is to synthesize the existing evidence and explore the applications of PE in rehabilitation. **Methods**: In line with Preferred Reporting Items for Systematic Reviews and Meta-Analyses guidelines, a systematic search was conducted across the PubMed, Web of Science, Scopus, and PEDro databases from inception to July 2025. The search strategy employed the term “Percutaneous Electrolysis” without applying additional filters or time restrictions, ensuring a comprehensive search. Cited references from screened articles were also evaluated for potential inclusion. Studies were included if they met the following criteria: peer-reviewed articles, intervention-based research, relevance to the topic, and publication in English. **Results**: Of the 181 papers retrieved, 143 were excluded for various reasons, leaving 38 studies. The evidence suggests that PE appears effective in reducing pain and improving function, particularly when combined with exercises such as eccentric training or stretching, though inconsistencies in protocols and patient characteristics, along with unclear mechanisms, show that it warrants further investigation. **Conclusions**: In conclusion, while PE emerges as a promising therapeutic strategy for musculoskeletal disorders, its full integration into rehabilitation practice necessitates further rigorous research to standardize treatment protocols, elucidate the underlying mechanism, and validate its cost-effectiveness. These steps are essential to establish PE as a robust and evidence-based option within the field of rehabilitation.

## 1. Introduction

Percutaneous electrolysis (PE) consists of the application of a galvanic current through an acupuncture needle placed into an affected soft tissue. Being applied with a very thin needle, PE is considered a minimally invasive technique and thus, albeit being fairly new, is easily accepted by patients [1]. PE serves as the overarching term that includes various techniques, such as Electrolysis Percutaneous Intratissue (EPI), percutaneous needle electrolysis (PNE—sometimes associated with higher-intensity currents) and Percutaneous Electrolysis Therapy (EPTE) [1]. EPI and EPTE are both specific methods within this category; EPI tends to use a higher intensity current while EPTE often employs a lower intensity current [1].

Ultrasound (US) guidance can be used when performing PE to achieve better therapy efficacy and to ensure that the target tissue is reached [2]. Solid scientific evidence is still lacking about PE; its effectiveness seems related to a local inflammatory response caused by a non-thermal electrolytic reaction that stimulates local repair and regeneration processes; a mechanical effect caused by needle insertion itself has been suggested, similarly to what happens in acupuncture [3,4]. Papers on murine models have shown an increased concentration of anti-inflammatory proteins and angiogenic factors alongside a decrease in pro-inflammatory mediators in tissues treated with PE. Moreover, genes related to collagen regeneration seem to have higher expression [5]. Another suggested that PE action has been related to a temporary vasodilation of healthy animal tendons: this could increase the removal of nociceptive molecule and pro-inflammatory cell arrival, a process crucial for tendon healing and regeneration [6]. PE’s ultimate goal is to generate an analgesic effect.

PE has been mostly applied for the treatment of musculoskeletal (MSK) pain, mostly related to tendinopathies, but the literature reports that other applications (e.g., mammary fistulas) are available, too [7,8]. MSK pain can manifest as either acute or chronic conditions. Acute MSK pain typically arises suddenly following trauma or overuse and is often self-limiting. In contrast, chronic MSK pain persists for more than three months, frequently resulting from failed tissue healing, repetitive microtrauma, or degenerative changes [9,10]. Most of the studies reviewed in this paper primarily address chronic MSK disorders (e.g., chronic tendinopathies such as patellar, Achilles, lateral epicondylitis, and plantar fasciitis) where pain and functional impairment are longstanding and resistant to initial conservative management. A smaller subset of studies investigates acute conditions, such as acute whiplash syndrome. The nature of MSK pain (acute or chronic) is an essential factor, as it influences both treatment strategies and the expected response to interventions.

Tendinopathies arise from a combination of factors, including impaired tendon healing, increased blood vessel formation (hypervascularization), and changes in the structure of collagen fibers and the surrounding matrix [11]. Among tendinopathies, patellar tendinopathy is an overuse injury predominantly affecting athletes involved in jumping sports, characterized by pain in the patellar tendon that can significantly impair athletic performance [12]. The injury is particularly prevalent in sports such as basketball and volleyball, where the repetitive loading of the knee is common; current treatment approaches for patellar tendinopathy emphasize conservative management, particularly focusing on load management and exercise therapy [13]. Epicondylitis primarily involves the extensor carpi radialis brevis tendon and is characterized by pain over the lateral epicondyle, affecting grip strength and overall function [14]. Supervised exercise that combines eccentric and static stretching was found to significantly outperform other conservative treatments, suggesting that tailored exercise regimens should be a cornerstone of non-operative management [15]. Subacromial pain syndrome is another common disease, characterized by pain localized around the acromion and often exacerbated by arm elevation. This syndrome includes various underlying etiologies, such as bursitis, supraspinatus tendinopathy, and partial rotator cuff tears [16]. Current treatment modalities for subacromial pain syndrome include nonsteroidal anti-inflammatory drugs and corticosteroid injections, which provide temporary relief by reducing inflammation in the subacromial space [17]. There is still a debate on the optimal treatment for tendinopathies, but physical therapy focused on managing tendon strain is usually the first approach. If pain lingers, other options like extracorporeal shock wave therapy or injections with several compounds (i.e., collagen, hyaluronic acid, or platelet-rich plasma (PRP)) injections might be explored, although their effectiveness can vary [18,19,20].

The management of MSK disorders typically involves a multimodal approach combining non-pharmacological, pharmacological, and, in some cases, surgical interventions. The main pillars include patient education and self-management, exercise therapy, pharmacological treatments (e.g., NSAIDs, acetaminophen, opioids), interventional procedures (e.g., injections), and psychosocial and behavioral therapies (to address the multidimensional nature of pain) [21,22,23,24,25,26,27,28,29]. PE is emerging as a new therapeutic approach whose effects, benefits, indications, optimal methods of application, and possible contraindications need to be understood more thoroughly. The primary aim of this systematic review is to explore the application of PE for the management of musculoskeletal disorders in the rehabilitation field. Secondarily, we aim to evaluate the most recent evidence in the literature about the various methodologies, rehabilitation protocol applied, US guide employment, and “the grey zones” of PE.

## 2. Materials and Methods

### 2.1. Search Strategy

This systematic review was performed in accordance with PRISMA 2020 guidelines [30]. A systematic search was performed in the online databases PubMed, Web of Science, Scopus, and PEDro until July 2025. The protocol for this systematic review has been duly registered with Open Science Framework registries accessible via the following registration link: https://doi.org/10.17605/OSF.IO/CMU9H. The literature search was structured according to the PICO framework (Population/Problem, Intervention, Comparison, and Outcome), as detailed in Table 1.

As a search strategy, the term “Percutaneous Electrolysis” was inserted for the search in all databases. No other specifier was applied, and no time limit was used, to keep the search as broad as possible. The cited references of the screened articles were assessed for potential inclusion. In order to provide a review that was as comprehensive as possible, case reports, case series, and cadaveric studies were included to enhance the depth and breadth of the research.

### 2.2. Screening Results and Eligibility

The inclusion criteria for this systematic review were determined through a structured process to ensure that only studies directly relevant to the research objectives were selected. The inclusion criteria were developed after clearly formulating the research question and specifying the objectives of the review, in accordance with PRISMA guidelines. Criteria were set prior to the formal literature search to minimize selection bias and ensure consistency throughout the screening process. Only peer-reviewed intervention studies were included. Studies had to involve patients with relevant MSK disorders, and no restrictions were placed on age, gender, or geographic location. Papers were also included if they involved the cadaveric evaluation of the potential application of PE. Studies were required to report on clinical outcomes relevant to MSK disorders, such as pain reduction, functional improvement, or safety/adverse effects. The articles were excluded if (1) the article was not peer reviewed; (2) the publication was a review article on the research topic; (3) the study was not an intervention; (4) the study was off topic; or (5) the paper was not in English.

Full-text manuscripts of accepted papers were retrieved and screened by two independent researchers (N.M. and A.S.); if there was a disagreement, a third author (C.P.) helped in the decision. After the selection, each title/abstract/full text was independently assessed by each of the authors. The extracted data were transcribed into standardized data collection sheets.

### 2.3. Data Extraction

Data concerning this topic were collected in an appropriate Excel (Microsoft, Redmond, Washington, DC, USA—version 16.99) spreadsheet and analyzed:

- General characteristic of the paper: first author, year of publication, study design.
- Study population characteristics. Human/no human, patients or healthy volunteers, age, gender, and type of disease.
- Methods: type of US imaging, setting; use of the US guide; operator performing the procedure; type of disease; rehabilitation protocol applied.
- Times during rehabilitation: pre-, during, post-, and follow-up.
- Outcomes and results.

### 2.4. Risk of Bias

Two researchers evaluated the study quality, and differences were solved after discussion. The papers were scrutinized for quality using Risk of Bias Assessment tool for Randomized Control Trials (RCTs). This tool includes different domains of bias: random sequence generation, allocation concealment, blinding of participants and personnel, blinding of outcome assessment, incomplete outcome data, selective reporting, and other bias. Each domain was judged as “low risk”, “high risk”, and “unclear”.

The case–control studies were assessed using Newcastle–Ottawa Scales (NOS) for case–control studies. The case-report studies were evaluated by Joanna Briggs Institute (JBI) Critical Appraisal Checklist for Case Reports for case-report studies. Animal studies were assessed using the Systematic Review Centre for Laboratory animal Experimentation (SYRCLE) Risk of Bias Table for Animal Studies, while cadaveric studies used the Quality Appraisal for Cadaveric Studies (QUACS) scale.

## 3. Result

**Figure 1:**
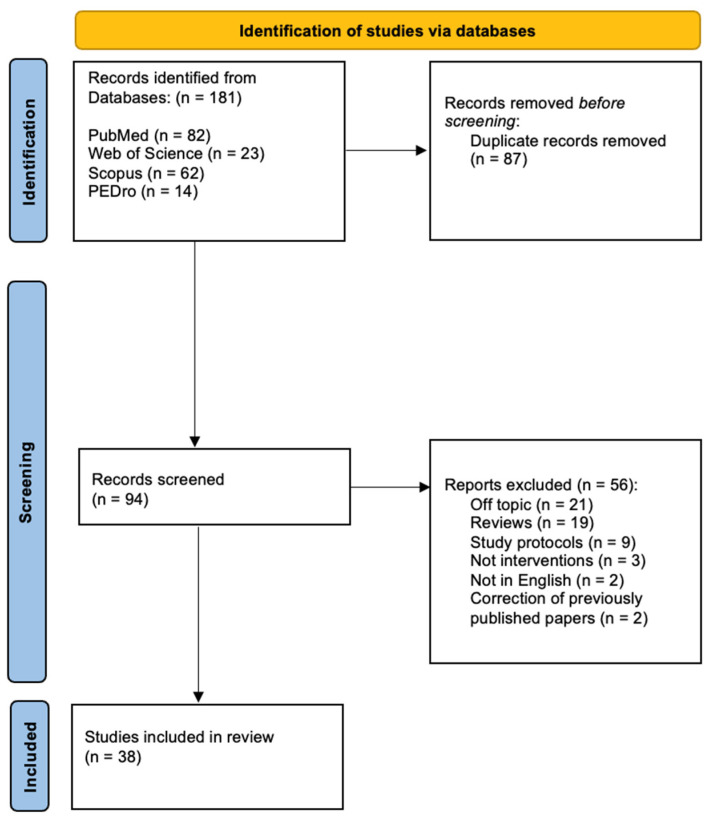
Study flowchart.

The study flowchart is shown in Figure 1.

A total of 181 papers were retrieved using the search methods described above. After duplicate (87) removal, 56 papers were excluded from this review after assessment because they were off topic (21), reviews (19), study protocols (9), not interventions (3), not in English (2), or corrections of previously published papers (2). Thirty-eight papers were thus included in this review and deeply analysed for the variables under examination: thirty-one were on MSK disorders, two were on animals, and five were cadaver studies.

### 3.1. Musculoskeletal Diseases

A wide heterogeneity has been observed regarding the application of PE in the field of musculoskeletal disorders. Among these papers, 29 (94%) were carried out on humans and 2 (6%) on animals (discussed in the relevant section). US guidance was used in 26 (84%) papers and was avoided in 1 (2%); the remaining 4 (14%) did not provide information about US application. A discrepancy was evident when sample sizes were analyzed with a wide overall range among the studies (min 8–max 102). The same result emerged when analyzing participants’ age, with an overall range of 16–62 years among the studies; the lowest reported mean age was 21.03, and the highest was 58 years. Of the 21 studies reporting operator data, physiotherapists (PT) performed the procedure in 19 (63%) cases, and 2 (7%) studies used the general term “clinician” without further details; 9 studies did not report the operator performing the procedure. Notably, only 11 (35%) papers reported data about adverse events, negative in 10 cases; 20 papers did not provide information on this topic.

In the assessed papers, the most represented pathology for PE use is patellar tendinopathy (six papers, 20%). Abat et al. [31] applied PE on 33 people (29 males, mean age 25.3 years) affected by insertional patellar tendinopathy. Patients received weekly sessions of PE and two weekly sessions of eccentric exercise; PE was applied through 3 milliamps echo-guided punctures (needles from 0.30 to 0.32 mm in diameter and a modified electric scalpel) until there was clinical improvement or no improvement in the symptomology was seen after 10 sessions; a two-year follow-up period was established. No adverse events were reported. Notably, like some of the other authors, Abat et al. applied a motor re-educational program (two weekly sessions of eccentric exercise) alongside PE and found that this combination is effective in reducing pain and improving functional status assessed via the Victorian Institute of Sport Assessment for the patellar tendon (VISA-P) scale. The same research group reported another protocol for patellar tendinopathy on 40 patients (35 males, mean age 25.5 years); a very similar PE application technique (one session every 2 weeks up to a maximum of ten sessions, with 0.3 mm in diameter acupuncture needles and intensity of 3 mA) alongside repeated sessions of eccentric exercise training. In this paper, Abat et al. followed up on patients for 10 years and confirmed that the treatment resulted in improvement in knee function assessed via the VISA-P scale and Tegner score [32]. Lopez-Rojo et al. [33] reported contrasting findings in their investigation comparing dry needling (DN) and PE with eccentric exercises for patellar tendinopathy. Forty-eight patients (forty-two males; mean age, 32.46 years) were divided into three groups: control (sham needling), DN, and PE (3 mA for 3 s). All groups performed eccentric exercises. A physiotherapist delivered the interventions. Outcomes were assessed using the VISA-P scale, the Visual Analog Scale (VAS) for pain, and Short-Form 36 (SF-36) for Quality of Life (QoL). The authors found that neither DN nor PE combined with eccentric exercises demonstrated greater efficacy than eccentric exercises alone in reducing pain and improving function at both short-term (10 weeks) and medium-term (22 weeks) follow-up. Notably, no adverse event was reported in the previous papers; however, two studies assessing the possible impact of PE on the autonomic system were retrieved. The first one, by de la Cruz Torres et al. [34], involved 22 male footballers (mean age 23.5 years) divided into two groups: the control (evaluation of heart rate variation at rest and during US examination of patellar tendon) and the experimental group (evaluation of heart rate variation at rest and during PE application at the patellar tendon by a PT-3 mA needle 0.3 mm). The experimental group showed increased parasympathetic and decreased sympathetic activity, implying the risk of vasovagal reaction. The same group published another paper in 2018 to assess if the aforementioned autonomic reaction could be related to the needle puncture alone or more specifically to PE. In this paper, 36 male footballers were recruited and allocated into three groups: control (evaluation of heart rate variation at rest and during US examination of the patellar tendon), PE group, and needle group (evaluation of heart rate variations at rest and during PE or needle puncture, respectively). The PE group showed significant autonomic reactions compared to the other two, suggesting a strong relation with electric current application [35]. Lastly, one paper analyzed the cost-effectiveness of three patellar tendinopathy treatments: PE, DN, or sham needling. The authors reported that the total cost per session was similar in the three groups, but the PE group presented better cost-effectiveness in terms of quality-adjusted life years [36]. One paper, by Valera-Carelo et al. [37], analyzed PE application on patellofemoral syndrome; 15 patients (mean age 25.6 years) were divided into three groups: high-intensity PE (660 mA for 10 s), low-intensity PE (220 mA for 30 s), and the DN control group. Both PE procedures seemed effective in reducing pain and caused less pain during interventions.

Another body area in which PE has been widely applied concerns foot disorders, particularly plantar fasciitis and plantar heel pain. Iborra-Marcos et al. [38] investigated the efficacy of PE compared to corticosteroid injections for plantar fasciitis in a study of 64 patients (35 males, mean age 46.4 years). Patients were divided into two groups: PE treatment (3 mA for 5 s, administered weekly for up to 10 sessions) or corticosteroid injections (1 mL mepivacaine and 1 mL betamethasone acetate+betamethasone sodium), both US-guided. The study did not report the healthcare professional performing the procedures. At 12-month follow-up, both interventions showed improvements in pain (VAS) and function (Foot and Ankle Disability Index—FADI). However, corticosteroid injections required fewer administrations and achieved superior outcomes in both pain and function.

Fernández-Rodríguez et al. [39] investigated the effectiveness of US-guided PE for chronic plantar heel pain. They recruited 73 patients and divided them into two groups: one receiving PE and a control group receiving a placebo puncture. The PE group underwent one session of cathodal PE (28 mC) per week for five consecutive weeks. The study focused on four main outcomes: pain, function, and disability (21-item activities of daily living subscale of the Foot and Ankle Ability Measure questionnaire), plantar fascia thickness measured by ultrasound, and all these were assessed at baseline (pre-treatment), 12 weeks, and 24 weeks. Patients in the PE group showed significantly better results in pain, function, and disability at both the 12-week and 24-week follow-ups compared to the control group. Al-Boloushi et al. compared the effectiveness of PE (1.5 mA) and DN for pain, function, and QoL in 102 patients (30 males, mean age 48.8 years) with plantar heel pain. All participants also received a stretching protocol. Both PNE and DN significantly reduced both mean and maximum pain scores from the first treatment session onwards, demonstrating long-lasting effects (up to 52 weeks). Notably, the PE group exhibited significantly better QoL at the 52-week follow-up compared to the DN group [40]. Building on their prior work, the same group conducted a cost-effectiveness analysis using the same data to compare PE and DN for plantar heel pain [41]. Their findings suggest that PE may be a more cost-effective approach compared to DN, particularly at the 52-week follow-up, where statistically significant differences in cost-effectiveness were observed.

A study by García Naranjo et al. [42] investigated the effectiveness of PE for acute whiplash syndrome. The researchers recruited 100 patients (36 males, average age 38.1 years) and divided them into two groups: PE and physiotherapy. The PE group received three weekly sessions of US-guided PE (4 mA) performed by a trained physiotherapist. The physiotherapy group received a combination of treatments including microwave thermotherapy, analgesic Transcutaneous Electric Nerve Stimulation (TENS) currents, massage, therapeutic ultrasound, active exercises, and stretching, all delivered daily for four weeks. Both groups showed similar improvements in pain and neck function as measured by the Northwick Park Neck Questionnaire and VAS. However, the PE group demonstrated significantly better results in pain pressure threshold. Notably, physiotherapy required a significantly longer treatment time, averaging 20 h compared to less than 1 h for the entire PE intervention.

Moreno et al. [43] investigated the effectiveness of PE for adductor longus enthesopathy-related groin pain in soccer players. They recruited 24 non-professional male athletes (men age 26.1 years). The participants were divided into two groups: US-guided PE (two sessions per week, 3 mA for 5 s) combined with active physiotherapy (APT), and a control group receiving only physiotherapy. The study followed the participants for six months. The combined intervention of PE and APT resulted in a greater and faster reduction in pain compared to physiotherapy alone. Additionally, the PE group showed a tendency towards better functional recovery, and these positive treatment effects persisted for at least six months.

Sanchez-Gonzalez et al. [44] investigated the potential effects of PE on endogenous pain mechanisms in a study of 46 asymptomatic participants (15 males, aged 18–40 years). The participants were divided into three groups: sham (PE application without electrical current), low-intensity PE (0.3 mA for 90 s), and high-intensity PE (three pulses of 3 mA, each lasting 3 s). All PE interventions were administered under US guidance by a physiotherapist. The researchers assessed widespread pressure pain thresholds, conditioned pain modulation, and temporal summation bilaterally in four specific areas: the lateral epicondyle, the bicipital groove, the C5 transverse process, and the tibialis anterior muscle. Their findings suggest that a single session of PE can mildly stimulate pathways involved in regulating pain perception (nociceptive gain). Interestingly, no significant differences were observed between the low-dose and high-dose PE applications. Varela-Rodriguez et al. [1] also conducted an RCT to investigate whether PE can induce endogenous pain modulation mechanisms and whether these effects are dependent on the dosage of the galvanic current. Fifty-four asymptomatic participants (thirty-four males, mean age 22.96 years) were enrolled and allocated into three groups, each receiving a single ultrasound-guided PE application on the lateral epicondyle tendon. The groups received either sham (without electrical current), low-intensity (0.3 mA, 90 s), or high-intensity (three pulses of 3 mA, 3 s) stimulation. Widespread pressure pain thresholds, conditioned pain modulation, and temporal summation were evaluated in the elbow, shoulder, and leg regions. The study found that a single PE intervention modulated pain processing in local and widespread areas, implying an endogenous pain modulation effect. Interestingly, this effect appeared to be independent of the dosage administered.

Varela-Garrido et al. [45] investigated the effectiveness of a multimodal program for chronic lateral epicondylitis in 36 patients (19 males, average age 38 years). The program combined US-guided PE, eccentric exercise, and stretching. The researchers measured patient outcomes at baseline, at discharge, and during follow-ups at 6, 26, and 52 weeks. All outcome measures showed significant improvements between pre-intervention and discharge, indicating that the multimodal program effectively reduced pain and improved the ultrasound-assessed structure of the tendons. Yildizgoren et al. [46] presented a case report examining the biochemical and ultrasound features of PE in a 45-year-old woman with chronic lateral epicondylitis who had not responded to conventional treatments. The patient received three sessions of US-guided PE (350 μA, 80 s per session). Pain and functional outcomes were assessed at baseline and four weeks post-treatment using the VAS and QuickDASH questionnaire. The patient’s VAS score improved from 8 to 2, and her QuickDASH score improved from 56 to 18. Ultrasound imaging during the procedure revealed hyperechoic gas formation, attributed to hydrogen gas generated by electrolysis. No adverse events were observed. These findings suggest that US-guided PE combined with exercise and stretching is beneficial for managing chronic lateral epicondylitis.

De-la-Cruz-Torres et al. [47] investigated the effectiveness of PE for chronic soleus injury in dancers. Thirty dancers (twenty-seven males, average age 21.03 years) were randomly assigned to one of three groups: PE, eccentric exercise, or a combined PE and exercise program (n = 10 per group). A PT administered the PE intervention, which consisted of two sessions of US-guided PE (one session per week) with an intensity of 2.5 mA, delivered for 3 s per application and repeated three times per session. The study found that the combined PE and exercise group showed a significantly greater improvement compared to the other groups. Dancers in the combined group also reported a greater perception of improvement.

Jimenez-Rubio et al. [48] carried out a protocol to validate a new, functional on-field program for the rehabilitation of soccer players after a hamstring strain injury. Nineteen professional male soccer players (mean age 24.23 years) were recruited for the purpose of this paper. Following the clinical diagnosis of a hamstring strain injury, players underwent PE followed by physiotherapy (mobilization and strengthening exercises). Subsequently, players participated in a progressive, on-field readaptation program consisting of 13 drills. The mean time to return to play was 22.42 ± 2.32 days.

Rodríguez-Huguet et al. [49] investigated the efficacy of PE on lateral epicondylalgia in two studies. The first study was an RCT enrolling 32 patients (20 males, mean age of 38.16 years) divided into two groups: a PE group (16) and a DN group (16). A PT performed the interventions four times, which were administered alongside a daily eccentric exercise program. Patients were followed up for up to three months. Significant between-group differences in pain and flexion movement were found after treatment, suggesting that PT may be more effective than DN for the short- and medium-term improvement of pain and function in lateral epicondylalgia. The second study compared an integrated intervention of pulsed negative-pressure myofascial vacuum therapy, PE, and eccentric exercise to a program of manual therapy mobilization, US therapy, and eccentric exercise. Forty participants (twenty-five males) with a mean age of 40.1 years were equally divided between the groups. The PE group received four weekly sessions, while the control group received ten sessions over two weeks. Both groups performed daily eccentric exercises at home. The PE group showed statistically significant improvements in pain intensity and function compared to the control group after treatment, with these benefits maintained at the one-month and three-month follow-up [50].

Benito-de-Pedro et al. [51] conducted an RCT to compare the short-term pain reduction effects of PE versus DN on myofascial trigger points of the levator scapulae in patients with chronic non-specific neck pain for at least 3 months. Fifty-two patients (sixteen males, mean age 38.77 years) were enrolled. Pain intensity, pressure pain threshold, cervical range of motion, and neck disability were assessed. Additionally, post-needling soreness was evaluated. The study found that PE and DN appear to have similar short-term effects on pain reduction, although PE was reported as a more painful treatment compared to DN.

Moreno et al. [52] investigated the feasibility of PE as a treatment for rectus abdominis-related groin pain in professional footballers in a pilot study. Eight professional male soccer players (mean age 26.8 years) participated in the study and received ultrasound-guided PE delivered by a PT. Participants received PE only; no other concurrent treatments were administered. Participants were followed up for up to 6 months. PE was delivered at 3 mA for 4 s during 2 to 6 sessions administered once weekly. PE treatment resulted in complete pain resolution within one month. Patients achieved excellent functional recovery for walking and jogging within one week, and for getting out of bed, running, jumping, and kicking within one month of treatment completion.

Another pilot study on professional footballers was the one by De-la-Cruz-Torres et al. [53]. The authors examined the effects of adding US-guided PE to a specific exercise program for soleus injury in female soccer players on perceived pain at stretching and at palpation, ankle dorsiflexion range of motion, muscle fatigue, and sport performance. Twenty female soccer players (mean age 22.5 years) were allocated into two groups: an experimental group receiving the exercise program combined with US-guided PE and a control group receiving the exercise program with sham stimulation. Both groups showed significant improvements in pain intensity (palpation and stretching), ankle dorsiflexion range of motion, and heel raise test values between pre-treatment and post-treatment. There were no significant differences in the curve sprint test results between pre-treatment and post-treatment within either group or between the groups. However, the percentage changes favored the experimental group, suggesting a potential benefit of PE that requires further investigation in larger studies with a higher statistical power.

Two papers were retrieved about subacromial pain syndrome. The first one, by de Miguel Valtierra et al. [54], was an RCT investigating the efficacy of US-guided PE as an adjunct to manual therapy and exercise in 50 patients (23 males; mean age 54 years) diagnosed with subacromial pain syndrome. Participants were allocated to two groups: one receiving manual therapy and exercise alone and another receiving the same intervention plus US-guided PE. The exercise program focused on pain, shoulder disability, function, and pressure sensitivity, delivered in one session per week for five consecutive weeks. Patients in the US-guided PE group received the intervention at each treatment session. Subjects receiving manual therapy, exercise, and US-guided PE demonstrated significantly greater improvements in shoulder pain and function compared to those receiving manual therapy and exercise alone at all follow-up assessments up to 6 months. Notably, the addition of US-guided PE did not yield significant differences in disability scores compared to the control group. However, significant improvements were observed for pain and function. This study is noteworthy for reporting adverse events associated with US-guided PE, with six patients in the intervention group experiencing transient muscle soreness following the first two treatments, which resolved spontaneously within 24–36 h. The second study, by Arias-Buría et al. [55], investigated the comparative effects of US-guided PE combined with an eccentric exercise program for the rotator cuff muscles in patients with subacromial pain syndrome. Thirty-six participants (nine males; mean age 58 years) were randomized into two groups: one receiving US-guided PE alongside an eccentric exercise program and another receiving the exercise program alone. The exercise program consisted of eccentric exercises performed twice daily for four weeks. Patients in the US-guided PE group received the intervention at each exercise session. Individuals receiving the combined intervention demonstrated greater improvement in shoulder pain compared to those in the exercise-only group. This improvement was statistically and clinically significant for pain, but the effect on function did not reach the minimal clinically different threshold.

Another common shoulder problem is supraspinatus tendinopathy; two papers were retrieved on the subject. Rodríguez-Huguet et al. [56] conducted a randomized controlled trial investigating the effectiveness of PE compared to DN for supraspinatus tendinopathy. Thirty-six patients (twenty-seven males, mean age 40.04 years) were assigned to receive either PE (one treatment per week for four weeks, 350 μA for 1.2 min) or DN (weekly session for four weeks at the upper trapezius muscle towards supraspinatus). Both groups also performed eccentric exercises. The PE group showed significantly greater improvements in pain and shoulder range of motion at the one-year follow-up compared to the DN group. Góngora-Rodríguez et al. [57] employed an RCT design to investigate the effects of a combined intervention on supraspinatus tendinopathy. The intervention included PE, percutaneous peripheral nerve stimulation, and eccentric exercises (3 sets of 10 repetitions of 3 exercises, twice a day for 4 weeks). Fifty participants (thirty-six males; mean age, 44.24 years) were allocated into two groups. One group received four weekly sessions of PE and PPNS, while the other group received ten sessions of TENS and US. The PE group demonstrated statistically significant improvements in pain, strength, supraspinatus electromyographic amplitude, US characteristics of the tendon (including echogenicity, thickness, and hypervascularization), and shoulder function.

Lopez-Martos et al. [58] conducted an RCT to evaluate the effectiveness of PE and DN for temporomandibular myofascial pain. Sixty patients (eight males; mean age, 38.8 years) participated in the study. Patients were assigned to one of three groups: PE, DN, or sham needling procedure. The PE group received low-dose electrical stimulation (2 mA for 3 s) delivered via a needle inserted in the lateral pterygoid muscle. The DN group received a deep needle puncture into the trigger point without any electrical stimulation. The sham group received a sham procedure where pressure was applied to the skin without needle penetration. All groups received treatment once a week for three consecutive weeks. Follow-up assessments were conducted up to 70 days after treatment. The authors reported that the PE group experienced significantly greater and faster pain relief and improvement in mouth opening compared to both the DN and SNP groups. Papers on PE applied for MSK diseases are reported on Table 2.

### 3.2. Animal Studies

Only two papers were retrieved about animal studies. Sanchez-Sanchez et al. (2020) [5] investigated the effects of PE on Achilles tendon tendinopathy in a mouse model. They divided fifteen mice into three groups: PE, DN, and a control group with no treatment. All groups received a single intra-tendinous collagenase injection to induce tendinopathy. The PE group received three weekly sessions of PE (3 mA, 4 s). Gene analysis revealed increased activity of genes involved in rebuilding collagen and the surrounding tissue in the PE group, suggesting that PE may promote healing. Margalef et al. investigated a mouse model of sciatic nerve entrapment to assess the potential therapeutic effects of PE. The researchers applied PE (1.5 mA for 3 s, repeated three times) near the site of nerve entrapment caused by induced fibrosis. After three weeks, they observed a near-complete recovery of the compound muscle action potential amplitude, suggesting that PE may have facilitated the release of the entrapped sciatic nerve [61]. Papers on PE applied on animal models are reported in Table 3.

### 3.3. Cadaveric Studies

Five papers were retrieved about PE application on cadavers (Table 4). Belón-Pérez et al. [62] conducted a study to investigate the accuracy and safety of PE targeting the supinator muscle. The study involved two parts: a human volunteer experiment and a cadaveric dissection analysis. In the human volunteer experiment, PE was applied to five healthy participants using US imaging to guide needle placement. The researchers assessed two different needle insertion approaches with the forearm in supination and pronation, both with the elbow extended. The cadaveric dissection analysis involved three Thiel-embalmed cadaver forearms. A needle was inserted into the supinator muscle with the surrounding tissues intact. The researchers then left the needle in place during dissection to confirm if the tip had reached the targeted muscle. The study findings demonstrated that PE achieved accurate needle placement within the supinator muscle in 100% of the trials, based on both US imaging in the human volunteers and visual confirmation during cadaveric dissection. Additionally, no punctures of the deep branch of the radial nerve bundle were observed in any of the needle insertions. Calderón-Díez et al. [63] investigated the safety and accuracy of PE targeting the interface between the Achilles tendon and Kager’s fat. The study employed a two-part design as well: a human volunteer experiment with US guidance and a cadaveric dissection analysis. In the human volunteer experiment, 10 healthy individuals (4 males; mean age, 45 years) participated. US imaging was used to guide needle placement during PE application at the targeted interphase. A 25 × 0.3 mm filiform solid needle was inserted from the medial to the lateral side, under the body of the Achilles tendon, approximately 5 cm from its insertion point on the calcaneus. The cadaveric dissection analysis involved 10 fresh cadaver legs. However, unlike the human experiment, US guidance was not employed for needle placement in the cadavers. The study concluded that PE can be safely performed at the Achilles tendon–Kager’s fat interphase if US imaging is used to guide needle placement. The same group investigated the safety of US-guided PE targeting the interface between the patellar tendon and Hoffa’s fat pad. The study utilized a two-part design: a human volunteer experiment with US guidance and a cadaveric dissection analysis. In the human experiment, 10 healthy volunteers (7 males, mean age 42 ± 10 years; 3 females, mean age 33 ± 8 years) participated. US imaging was used to guide needle placement during PE application at the targeted interphase. A 25 × 0.3 mm filiform solid needle was inserted from the lateral to the medial side, traversing the patellar tendon to reach the deep interface with Hoffa’s fat pad. The procedure was performed by an experienced PT. The cadaveric analysis involved 10 knees from 5 fresh cadavers (2 males, mean age 69 ± 4 years; 3 females, mean age 75 ± 6 years). Unlike the human experiment, US guidance was not employed for needle placement in the cadavers. Dissection was performed to identify the anatomical relationship between the patellar tendon, saphenous nerve, and infrapatellar nerve branches. The study found that in all cadaveric specimens, infrapatellar nerve branches ran predominantly through the medial aspect of the knee and crossed in front of the patellar tendon. None of the nerve branches were pierced when the needle was inserted from the lateral side. In the human experiment, US imaging allowed for safe needle placement, though visualization of the infrapatellar nerve was challenging. The authors concluded that PE can be safely performed at the patellar tendon–Hoffa’s fat pad interface if a lateral approach is used and US imaging guides needle placement. The medial approach poses a higher risk to the infrapatellar nerve branches due to their variable anatomy and proximity to the tendon [64].

Borrella-Andrés et al. [65] conducted a cadaveric study to investigate whether PE applications generate temperature changes in musculoskeletal tissues. This research aimed to clarify a proposed mechanism of action for PE, which is its potential thermal effect. Ten cryopreserved knees (five from male donors and five from female donors) were used in the study. US guidance ensured accurate needle placement within the targeted tissues: patellar tendon, infra-patellar fat pad, and vastus medialis muscle. A thermometer was positioned near the needle tip to measure temperature changes during PE application. Two different PE protocols were employed depending on the targeted tissue: tendon: three applications of 3 s each with a current intensity of 3 mA; fat or muscle: three applications of 3 s each with a current intensity of 1.5 mA; all tissues: a single application of 24 s with a current intensity of 1 mA. The study found no significant temperature increases within the tendon, fat, or muscle tissues following PE application using either protocol.

Malo-Urriés et al. [66] conducted a cadaveric study to evaluate the dose-dependent structural effects of PE on muscle tissue using quantitative US imaging. The primary aim was to determine whether varying intensities of galvanic current induce measurable changes in muscle architecture and to identify objective ultrasound-based biomarkers for optimizing PE dosage. Twenty-nine samples of medial gastrocnemius muscle, obtained from cryopreserved cadavers (50% male, mean age 73.7 years), were used. Each sample received a single, randomly assigned dose of galvanic current ranging from 0 to 10 mA for one second under ultrasound guidance by an experienced operator. Quantitative US analysis was performed immediately after each application to assess geometric and textural changes in the tissue. The study found clear dose–response relationships in several ultrasound parameters, particularly the number, area, and perimeter of affected regions, as well as measures of homogeneity and contrast. Notably, significant structural changes became apparent at intensities above 1 mA, with a plateau effect observed beyond 4 mA, suggesting a physiological threshold for muscle tissue response.

### 3.4. Ultrasound Imaging and Percutaneous Electrolysis

The use of US guidance in PE was evident in all the papers and was motivated by several factors that enhance the safety, precision, and overall effectiveness of the treatment. US allows for the real-time visualization of the treatment area, enabling precise needle placement within pathological tissue. This precision is critical for the effectiveness of percutaneous electrolysis, as it ensures that the galvanic current is delivered exactly where it is needed to stimulate the desired healing response. Moreover, the ability to accurately target the damaged tissue minimizes the risk of affecting surrounding healthy structures, which can reduce the likelihood of unnecessary damage and improve patient outcomes. Additionally, US guidance was particularly useful in complex cases [31,32,45,48,52], where the anatomical structures are difficult to navigate without visual assistance. It allows practitioners to adapt the treatment to the specific needs of the patient, ensuring that the therapy is both effective and safe.

### 3.5. Risk of Bias Assessment and Applicability Concerns

**Figure 2:**
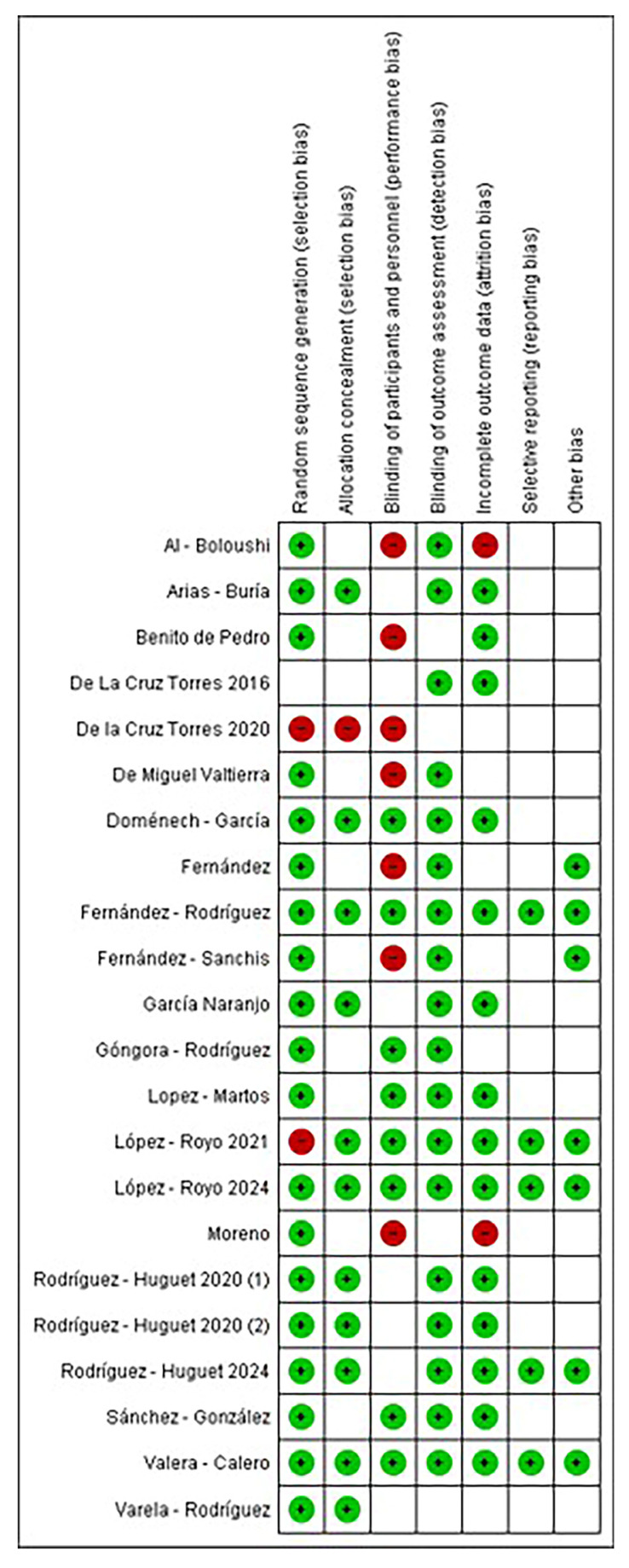
Risk of bias summary: review authors’ judgements about each risk of bias item for each included study. Green dots with “+”: low risk of bias; red dots with “−”: high risk of bias; white square: unclear risk. Al-Boloushi [40], Arias-Buría [55], Benito-de-Pedro [51], De la Cruz Torres (2016) [34], De la Cruz Torres (2020) [47], De Miguel Valtierra [54], Doménech-García [60], Fernández [41], Fernández-Rodriguez [39], Fernández-Sanchis [36], García Naranjo [42], Góngora-Rodríguez [57], Lopez-Martos [58], López-Royo (2021) [33], López-Royo (2024) [59], Moreno [43], Rodríguez–Huguet (2020-1) [49], Rodríguez-Huguet (2020-2) [56], Rodríguez-Huguet (2024) [50], Sánchez–González [44], Valera–Calero [37], Varela–Rodríguez [1].

The complete set of randomized controlled trials (RCTs) (k = 100%) was evaluated, and the scores are shown in Figure 2.

The 80% were assessed with a low risk of bias for random sequence (selection bias), and the 55% were assessed with low risk of bias for allocation concealment (selection bias). About the blinding of participants and personnel (performance bias), 40% were assessed with low risk, while 30% had a high risk and the other 30% had an unclear risk. Regarding the blinding of outcome assessment (detection bias), 80% were assessed with a low risk of bias and 20% with an unclear risk. Concerning incomplete outcome data (attrition bias), 70% were assessed with a low risk, 10% with a high risk, and 20% with an unclear risk. In relation to selective reporting (reporting bias), 25% were assessed with a low risk and 75% with an unclear risk. Finally, for other bias, 25% were assessed with a low risk, and 75% were assessed with unclear risk. NOS scores of the included studies’ articles are shown in Table 5. After evaluation by two researchers, the studies received an average NOS score of 9.0, indicative of high-quality studies.

The JBI Critical Appraisal Checklist for Case Reports Studies was used to assess the quality of case reports (Table 6).

The RoB for the included animal studies was evaluated using the SYRCLE Risk of Bias tool (Table 7). Sequence generation and allocation concealment were frequently not adequately addressed. Sánchez-Sánchez did not report random sequence generation or allocation concealment, indicating a high or unclear risk of selection bias. Margalef reported appropriate sequence generation but not allocation concealment [61]. Both studies lacked random housing and blinding of caregivers or investigators, suggesting a high risk of performance bias. Neither study reported random outcome assessment or blinding of outcome assessors, indicating a high risk of detection bias. Both studies adequately addressed incomplete outcome data, minimizing the risk of attrition bias. Both studies were free from selective outcome reporting, suggesting low risk in this domain.

The QUACS scale’s results are reported in Table 8. The main sources of potential bias across all studies were the lack of control for intra- and inter-observer variability and the absence of sample size justification. These limitations should be considered when interpreting the findings. However, the studies were otherwise methodologically sound, with clear aims, well-described procedures, and appropriate statistical analyses.

## 4. Discussion

PE is gaining traction as a novel therapeutic strategy for MSK disorders, particularly tendinopathies. Notably, the reviewed studies did not consistently report the inclusion of a motor re-education program alongside PE interventions. When present, motor re-education was typically implemented either concurrently with PE or immediately following the PE protocol, often continuing a previously established exercise regimen. Interestingly, we did not identify any data on the potential benefits of applying physiotherapy interventions specifically before PE treatment. The studies that incorporated motor re-educational programs primarily focused on eccentric exercises or stretching techniques. The association of PE and physiotherapy seems to improve the results of both techniques carried out alone. Standardized re-educational programs are crucial to ensure patient safety and proper technique.

In the course of our research, we found other possible delivery methods or diseases where PE is applied in different fields, either as protocols/suggestions or as completed clinical trials (i.e., for endogenous pain modulation [67], mammary fistulas [8,68], biceps brachii tendinopathy [69], debridement of the plantaris tendon [70], proximal hamstring tendinopathy-related sciatic nerve entrapment [71], patellar tendinopathy [72,73], plantar heel pain [74], chronic masticatory myalgia [75], infectious diseases [76,77], lung tumors [78,79,80], liver tumors [81], orbital venous malformation [82], to dissolve metallic stents leaving the luminal tissues intact [83], and recanalization of urinary collecting system obstructions [84]).

While PE application is gaining traction in clinical practice, several key points warrant further discussion based on the findings presented in this review. The studies included in this review suggest that PE may be effective in treating various MSK conditions, including patellar tendinopathy, plantar fasciitis, chronic low back pain, lateral epicondylitis, and others. However, it is important to note that a major limitation across the studies reviewed is the lack of standardized protocols for PE application. This inconsistency includes variations in participant characteristics (e.g., anthropometrics), evaluation methods, use of US guidance, and the incorporation of motor re-education. The absence of standardization makes it challenging to directly compare the findings and weakens the overall quality of evidence for PE. Another limitation identified in the reviewed studies is the lack of reporting on the healthcare professional administering PE. While the studies included here primarily involved PTs performing the procedure, not all studies disclosed this information. It is well-established that PTs play a vital role in rehabilitation of patients with MSK disorders through physiotherapy techniques like motor re-education and physical agent application [23,85]. However, concerns arise regarding the safety of PTs administering invasive or minimally invasive procedures like PE. Research suggests that PE can induce significant autonomic imbalance and potentially trigger vasovagal reactions [34,35]. While the clinical relevance of this finding requires further investigation, it highlights the importance of minimizing the risk of vasovagal episodes, including syncope. Therefore, to ensure patient safety, it is crucial for either a medical doctor to perform PE directly or for PTs to be adequately supervised by a physician during the procedure. Moreover, from a legislative standpoint, the practice of US-guided percutaneous needle electrolysis by physical therapists varies significantly depending on the country and its specific regulations governing physical therapy and invasive procedures.

The precise mechanisms underlying the therapeutic effects of PE remain elusive. Proposed theories suggest that PE may induce a localized inflammatory response, promoting tissue healing and regeneration [86]. Additional mechanisms might involve thermal changes, alterations in blood flow patterns, modulation of pain perception, or direct mechanical effects from needle insertion, similar to DN. However, in-depth analysis of PE’s effects on biological tissues is still lacking. Notably, one cadaveric study included in this review found that two different PE protocols did not generate significant temperature changes in tendon, fat, or muscle tissues. This suggests that a thermal effect is unlikely to be a primary mechanism of action for PE in clinical practice [65]. These findings are in agreement with an in vitro study by Margalef et al. [87] aimed at the evaluation of safety of PE procedures by analyzing potential alterations in the needles used. Authors examined the effects of three different PE protocols on commonly used PE needles. Temperature changes were measured by immersing the needles in test tubes containing Ringer’s solution. A multimeter assessed changes in electrical resistance, and a scanning electron microscope was used to compare the pre- and post-treatment needle morphology. Additionally, radiographic diffusion analysis evaluated the needle composition. The study found no significant temperature changes in Ringer’s solution during PE application. Notably, the in vitro analysis did not detect any loss of metal particles or alterations in the needle morphology following PE protocols, which is reassuring for the application of PE in clinical practice.

This review also identified a wide heterogeneity in treatment protocols across studies, including variations in needle type, intensity of electrical current, and treatment duration. Standardization of treatment protocols is necessary to optimize PE’s effectiveness and facilitate comparisons between studies. PE administration under US guidance seems to be a valid option to raise its effectiveness and safety. US-guided invasive procedures are usually more precise, as also confirmed by cadaver studies [4,62,73]. US provides real-time visualization, allowing for precise needle placement within pathological tissue, which is crucial for delivering the galvanic current effectively and minimizing harm to surrounding healthy structures. This approach is effective in reducing pain and enhancing the combination with other rehabilitation techniques.

As previously mentioned, these studies were conducted using varying protocols. Additionally, discrepancies in terminology and the explanation of methodologies pose a significant limitation, making it difficult to translate and compare results. This extreme heterogeneity hampers to the performance of quantitative analyses and undermines the overall level of evidence. Moving forward, it is crucial to develop more high-quality clinical trials to validate the use of PE in the different musculoskeletal disorders and facilitate the application in daily practice, ensuring that these key technical aspects are standardized. Lastly, cost-effectiveness analyses suggest PE might be a cost-effective option for some MSK conditions compared to other interventions. However, further research is needed to confirm these findings.

Importantly, PE aligns with the principles of personalized medicine (PM), which emphasizes tailoring treatments to individual patient profiles [88]. The variability in patient responses to PE underscores the potential for precision approaches, such as customizing galvanic current intensity, needle placement, and treatment frequency based on specific conditions and individual characteristics. Gender differences should also be systematically considered to evaluate potential variations in outcomes and responses [89]. By integrating PM into PE protocols, clinicians can deliver targeted interventions that maximize therapeutic benefits while minimizing risks. This personalized approach could significantly enhance the clinical utility of PE in rehabilitation settings.

### Limitations and Future Perspectives

Our findings showed that PE emerged as a clinically meaningful tool to improve pain and functional outcomes for tendinopathies and other musculoskeletal disorders, particularly when combined with eccentric exercise or stretching protocols. However, heterogeneity in treatment parameters (e.g., current intensity: 0.3–660 mA, session frequency: 1–10 sessions), operator expertise, and patient selection criteria limit its standardization. While most of the assessed studies used US guidance to enhance precision, significant gaps persist in understanding its biological mechanisms and long-term cost-effectiveness. Overall, the included studies showed low bias levels, but sample size was generally fairly small.

Thus, current evidence supports the use of PE as a supportive intervention, but it should not yet be recommended as a first-line standard treatment. The reason for this is the lack of comparative effectiveness data, protocol variability, and uncertainty about PE’s mechanistic effects. PE may play a role in multimodal Individual Rehabilitation Projects [90,91,92] for the treatment of patients with chronic tendinopathies unresponsive to physical therapy and athletes needing an accelerated return to play. Patient selection criteria, protocol standardization, and practitioner training requirements are central for clinical integration. Contraindications to be considered should involve acute inflammation, cardiac pacemakers, autonomic dysfunction, and local skin infections. Based on current evidence, a current intensity of 1.5–3 mA, with sessions occurring once a week for 3–5 weeks, not exceeding ten sessions, appears as the best clinical framework. Adjunctive measures include eccentric exercises and US guidance. Practitioner training requires physicians (physiatrists or orthopedic surgeons), along with safety protocols for autonomic instability screening and post-treatment monitoring. Future research priorities should include studies on electrolysis-induced cellular changes, dose–response RCTs comparing low- and high-intensity protocols, and health economic analyses to assess cost-effectiveness against other established therapies.

## 5. Conclusions

PE is emerging as a novel and promising treatment for MSK disorders, especially those affecting tendons. Studies show PE effectively reduces pain and improves function in several conditions. US guidance is often used for accurate needle placement and better results. Interestingly, most studies combined PE with physiotherapy exercises, typically eccentric exercises or stretching, and this combination seems to be more effective than PE alone. However, these physiotherapy programs were inconsistently applied, either alongside PE or following PE, highlighting the need for standardized re-educational programs to ensure patient safety and proper technique. By integrating PM principles into PE protocols, more targeted and effective interventions could be delivered while minimizing risks. This personalized approach could significantly enhance PE’s clinical utility in rehabilitation settings. Some points still raise concerns: variations in patient characteristics, treatment methods, and even who administers PE make it difficult to compare results and assess the overall effectiveness of PE; secondly, the exact mechanisms by which PE works are still unclear; thirdly, cost-effectiveness analyses suggest PE might be a cost-effective option, but more studies are needed to confirm this. In conclusion, PE is a promising new approach for treating MSK disorders, but further research is necessary to standardize protocols, understand its mechanisms of action, and confirm its cost-effectiveness.

## Figures and Tables

**Table 1:** Description of the PICO (P = Population, I = Intervention, C = Comparison, O = Outcome) elements.

Population	Patients or Healthy Volunteers who Underwent Percutaneous Electrolysis for Musculoskeletal Diseases
Intervention	Percutaneous Electrolysis
Comparison	Not applicable
Outcome	Pain, function, mobility, thickness, quality of life, and ability to reach the target tissue.

**Table 2:** Papers on the use of percutaneous electrolysis for management of musculoskeletal disorders.

Authors, Year	Title	Study Type	Human/Not Human	US Imaging	Sample Size	Sex	Age: Mean, (Range)	Operator	Type of Disease	Rehabilitation Pre	Rehabilitation During	Rehabilitation Post	Follow-Up	Setting	Outcomes	Results	Adverse Events
**Ferran Abat, 2014** [31]	Effectiveness of the Intratissue Percutaneous Electrolysis (EPI^®^) technique and isoinertial eccentric exercise in the treatment of patellar tendinopathy at two years follow-up	Prospective Case Series	Human	YES	33	M: 29; F: 4	25.3 years (16–53)	/	patellar tendinopathy	not	Two weekly sessions of eccentric exercise using isoinertial resistance machines	/	up to two years	Weekly session of EPI^®^ and two weekly sessions of eccentric exercise. Patients received the intratissue percutaneous electrolysis (EPI^®^) technique treatment until there was clinical improvement or no improvement in the symptomology was seen after 10 sessions, 3 milliamps echo-guided punctures, needles of from 0.30 to 0.32 mm in diameter and a modified electric scalpel	Victorian Institute of Sport Assessment for the patellar tendon (VISA-P), the Tegner scale, Roles and Maudsley scale	Average 35 points improvement in VISA-P	No adverse events
**Ferran Abat, 2015** [32]	Clinical results after ultrasound-guided intratissue percutaneous electrolysis (EPI^®^) and eccentric exercise in the treatment of patellar tendinopathy.	Prospective Case Series	Human	YES	40	M: 35; F: 5	25.5	/	Patellar Tendinopathy	NOT	YES (2 weekly sessions of eccentric exercise training using the resistance isoinertial leg-press machine)	NOT	3 months, 2 years, 5 years, 10 years.	Session of PNE every 2 weeks up to a maximum of ten sessions. Acupuncture needles (0.3 mm in diameter) with different lengths. Intensity 3 mA	Victorian Institute of Sport Assessment–Patella (VISA-P); Tegner score; the Roles and Maudsley score	Treatment with the US-guided EPI technique and eccentric exercises in patellar tendinopathy resulted in a great improvement in knee function and a rapid return to the previous level of activity after few sessions	No adverse events
**Fermín Valera-Garrido, 2014** [45]	Ultrasound-guided percutaneous needle electrolysis in chronic lateral epicondylitis: short-term and long-term results	Prospective Case Series	Human	YES	36	M: 19; F: 17	38 ± 6.4	/	chronic lateral epicondylitis	NOT	Yes (home program consisting of eccentric exercise and stretching)	NOT	6, 26 and 52 weeks	Session of PNE per week over 4 weeks. Intensity of 4–6 mA for 3 s approximately three times. A 0.3 × 25 mm (1 inch) acupuncture needle	VAS, DASH, US evaluation, patients’ perceptions of the overall outcome	Symptoms and degenerative structural changes of chronic lateral epicondylitis are reduced after US-guided PNE associated with EccEx and stretching	No adverse events
**José L Arias-Buría, 2015** [55]	Ultrasound-Guided Percutaneous Electrolysis and Eccentric Exercises for Subacromial Pain Syndrome: A Randomized Clinical Trial	RCT	Human	YES	36, randomly assigned into US-guided PE (n = 17) group or exercise (n = 19) group	M: 9; F: 27	58 ± 7	clinician	Subacromial Pain Syndrome	/	Eccentric exercise program of the rotator cuff muscles	/	1 week	Application of galvanic current through acupuncture needle on each session once a week (total of 4 sessions), acupuncture needles of different lengths (0.3 mm in diameter, intensity of 350 μA for 1.2 min)	Shoulder pain (NPRS) and disability (DASH)	US-guided percutaneous electrolysis combined with eccentric exercises resulted in slightly better outcomes in the short term compared to when only eccentric exercises were applied in subacromial pain syndrome	No adverse events
**Blanca de la Cruz Torres, 2016** [34]	Autonomic responses to ultrasound-guided percutaneous needle electrolysis of the patellar tendon in healthy male footballers.	RCT	Human	YES	22	M: 22	23.5	PT	patellar tendon in healthy male footballers	NOT	/	NOT	NOT	One session of PNE. Intensity of 3 mA. Acupuncture needles with 0.3 mm diameter and different lengths	Personal Psychological Apprehension Scale (PPAS), heart rate variability (HRV), the standard deviation of the RR intervals (SDNN), the square root of the mean of the sum of the squares of the differences between the adjacent RR intervals (rMSSD), and the number of adjacent RR interval (RRI) pairs that differ by >50 ms in the full register, divided by the total number of RRIs and expressed as a percentage (pNN50). The reverse axis (SD1), the longitudinal axis (SD2)	Significant increase in parasympathetic activity (in keeping with a potential vasovagal reaction) during application of the US-guided PNE technique on healthy patellar tendons of male football players	Measurable increase in parasympathetic activity (detected by HRV)
**Carlos Moreno, 2016** [52]	Therapeutic results after ultrasound-guided intratissue percutaneous electrolysis (EPI^®^) in the treatment of rectus abdominis-related groin pain in professional footballers: a pilot study.	Prospective Case Series	Human	YES	8	M: 8	26.8	PT	rectus abdominis-related groin pain	NOT	NOT	NOT	24 h, 1 week, 1 month, 6 months	Four (from 2 to 6) sessions, once a week, with a needle 0.25 × 30 mm in diameter. Intensity of 3 mA for 4 s	Verbal Rating Scale (VRS), Patient-Specific Functional Scale	Treatment with ultrasound-guided EPI has shown encouraging clinical results for RAGP.	N.A.
**Carlos Moreno, 2017** [43]	Intratissue percutaneous electolysis combined with active physical therapy for the treatment of adductor longus enthesopathy-related groin pain: a randomized trial	RCT	Human	YES	24	M: 24	26.1	PT	Adductor longus enthesopathy-related groin pain (ALErGP)	NOT	YES	NOT	6 months	Two sessions a week of PNE. Intensity of 3 mA. A 0.33 × 50 mm in diameter of s acupuncture needle. Intensity of 3 mA. 3 applications every session (3 right + 3 left if the ALErGP- adductor longus enthesopathy-related groin pain was present bilaterally), with a duration of 5 s each	Patient-Specific Functional Scale (PSFS), Numeric Rating Scale (NRS)	PE treatment in association with active physiotherapy ensured a greater and more rapid reduction of pain and tended to promote greater functional recovery in soccer players with ALErGP compared to active physiotherapy only.	No adverse events
**García Naranjo J, 2017** [42]	A novel approach in the treatment of acute whiplash syndrome: ultrasound-guided needle percutaneous electrolysis. A randomized controlled trial.	RCT	Human	YES	100, divided in two groups: physiotherapy and PE	M: 36; F: 64	38.1	PT	acute whiplash syndrome	NOT	NOT	NOT	5 Weeks	Weekly session for three weeks (3 sessions) with 25 × 0.16 mm acupuncture needles. Starting intensity was 2 mAmp, which was increased on a 1 mAmp/s speed to reach 4 mAmp, repeated three times per session, with a resting 210 interval of 1–2 min between shocks.	VAS, Northwick Park Neck Questionnaire (NPQ), pressure pain 167 threshold (PPT) with algometric assessment	Patients receiving the therapy substantially decreased their pain, pressure-pain threshold and quality-of-life measures, equally to standardized physiotherapy programs. Distinguishly, PNE protocol consists of only 3 application sessions of 15 min each one, with no added interventions	N.A.
**Lorena de Miguel Valtierra, 2018** [54]	Ultrasound-Guided Application of Percutaneous Electrolysis as an Adjunct to Exercise and Manual Therapy for Subacromial Pain Syndrome: A Randomized Clinical Trial	RCT	Human	YES	50 randomized into manual therapy/exercise (n = 25) or the manual therapy/exercise plus electrolysis (n = 25)	M: 23; F: 27	54 ± 7 years	PT	Subacromial Pain Syndrome	/	manual therapy and exercise: the program consisted of 3 exercises focusing on supraspinatus, infraspinatus, and scapular stabilizer muscles. Each exercise was performed in 3 sets of 12 repetitions	During the follow-up period, participants were asked to continue with the exercise program and this was monitored on subsequent follow-up assessments.	Up to 6 months	0.30 mm × 25 mm needle, intensity of 350 µA for a total of 90 s	Disabilities of the Arm, Shoulder and Hand (DASH) questionnaire. Secondary outcomes included pain, function (Shoulder Pain and Disability Index [SPADI]) pressure pain thresholds (PPTs) and Global Rating of Change (GROC)	The inclusion of US-guided PE in combination with manual therapy and exercise resulted in no significant differences for related disability compared with the application of manual therapy and exercise alone in patients with subacromial pain syndrome. Nevertheless, differences were reported for shoulder pain and function	No adverse events
**Álvaro Iborra-Marcos, 2018** [38]	Intratissue Percutaneous Electrolysis vs. Corticosteroid Infiltration for the Treatment of Plantar Fasciosis	Retrospective Case-Control	Human	YES	64 patients: 32 treated with ultrasound-guided EPI and 32 with ultrasound-guided Corticosteroid infiltration	M: 35; F: 29	46.4 ± 8.5	/	plantar fasciosis	/	/	/	1 year	G32 needle, 3 mA current was delivered for 5 s. The treatment was repeated 7 days later and then again for up to 10 sessions at weekly intervals as required	Visual analog scale (VAS) to record pain and the Foot and Ankle Disability Index (FADI)	Both techniques were effective in the treatment of PF, providing excellent VAS pain and FADI results at 12 months. However, CI required fewer patient visits and appeared to provide somewhat better VAS and FADI results	No adverse events
**Ricardo Lopez-Martos, 2018** [58]	Randomized, double-blind study comparing percutaneous electrolysis and dry needling for the management of temporomandibular myofascial pain	RCT	Human	NOT	60	M: 8; F: 52	38.8 (18–62)	/	temporomandibular myofascial pain	NOT	/	YES (two weeks after each procedure, concentric exercises with the masticatory muscles)	28, 42, and 70 days	Session of PNE once per week, for 3 consecutive weeks. Intensity of 2 mA for 3 s. A 0.25 × 40 mm acupuncture needle	VAS, maximum interincisal opening (MIO) without causing pain or discomfort, involvement of the TMJ, assessed by a 100-point questionnaire, Tolerability to the treatment was evaluated by the patient and the observer using a 5-point scale, ranging from 0 (very bad) to 4 (excellent)	greater and earlier relieving pain and improving MIO of patients treated with percutaneous needle electrolysis compared to deep dry needling and sham needling procedure	No adverse events
**Paula Garcìa Bermejo, 2018** [35]	Autonomic Responses to Ultrasound-Guided Percutaneous Needle Electrolysis: Effect of Needle Puncture or Electrical Current?	Prospective Case–Control	Human	YES	36	M: 36	24.36	PT	patellar tendinopathy	NOT	NOT	NOT	NOT	One session of PNE, with three applications with needles with 0.3 mm diameter and different lengths. Intensity of 3 mA for 3 s	Personal Psychological Apprehension Scale (PPAS), diameters of the Poincare’s plot (SD1, SD2), stress score, and sympathetic/parasympathetic ratio	The application of the US-guided PNE technique caused a measurable increase in parasympathetic activity (detected by heart-rate variability—HRV), which was due to the combination of needle puncture and electric current	Significant Autonomic Imbalance (In Keeping with A Potential Vasovagal Reaction)
**Fernández-Rodríguez T, 2018** [39]	Prospective Randomized Trial of Electrolysis for Chronic Plantar Heel Pain.	RCT	Human	YES	73 (PE group or placebo puncture)	M: 31, F: 42	46	Clinician	chronic plantar heel pain	NOT	/	YES	1, 12, and 24 weeks	Session of PE once per week, for 5 consecutive weeks. A 0.35 × 40 mm acupuncture needle. Intensity of 28 mC of cathodal PNE.	VAS, 21-item activities of daily living subscale of the Foot and Ankle Ability Measure questionnaire, and plantar fascia thickness measured by ultrasound.	Improved pain and function. This treatment may also decrease fascia thickness (but further studies are needed for this last point).	No adverse events
**Jiménez-Rubio S, 2019** [48]	Validity of an On-Field Readaptation Program Following a Hamstring Injury in Professional Soccer.	Prospective Case Series	Human	YES	19	M: 19	24.23 ± 5.36	/	Hamstring Injury	NOT	YES	/	/	/	Aiken’s V for each item of the program and number of days taken by the players to return to play	The program proposed for the rehabilitation and readaptation phase following an injury to the hamstring muscle complex was determined to be valid by the panel of experts, given its soccer-specific context and that the entire program was carried out on the field.	N.A.
**Manuel Rodríguez-Huguet, 2020** [49]	Percutaneous Electrolysis in the Treatment of Lateral Epicondylalgia: A Single-Blind Randomized Controlled Trial	RCT	Human	YES	32: trigger point dry needling (n = 16) and PE group (n = 16)	M: 20; F: 12	38.16 ± 13.89	PT	Lateral Epicondylalgia	/	Eccentric exercise program to be performed daily (three series of ten repetitions of eccentric work twice daily (morning and afternoon) with 1 kg weights)	/	up to three months	EPTE^®^ percutaneous electrolysis device (Ionclinics & A. Deionic SL, Valencia, Spain) for 1.2 min at an intensity of 350 µA in the insertional tendon of the muscles of the epicondyle using a 0.3 mm needle guided by ultrasound (Voluson 730 pro, General Electric^®^, Boston, MA, USA) and forming an angle of between 30° and 45° with the axis. The treatment was performed once a week for four weeks	Numerical pain rating scale (NPRS), pressure pain thresholds (PPT), SF-12, and elbow range of motion	Ultrasound-guided percutaneous electrolysis as an adjunct to an eccentric exercise program is more effective for pain and range of movement than trigger point dry needling	No adverse events
**Manuel Rodríguez-Huguet, 2020** [56]	Effectiveness of Percutaneous Electrolysis in Supraspinatus Tendinopathy: A Single-Blinded Randomized Controlled Trial	RCT	Human	YES	36: PE group (n = 18) or a trigger point dry needling group (n = 18)	M: 27; F: 9	25–60 (40.04 ± 9.88)	/	Supraspinatus tendinopathy	/	Eccentric exercises for the supraspinatus to be performed daily at home from the first to the last day of treatment (3 × 10 repetitions):	/	1 year	PE group: One treatment per week over four weeks (four sessions in total) using a percutaneous electrolysis EPTE^®^ device (Ionclinics & Deionic S.L., Valencia, Spain) at an intensity of 350 μA for 1.2 min was performed. PE was applied on the injured zone of the supraspinatus tendon, which was located by ultrasound Trigger point dry needling group: A weekly session for four weeks (four sessions in total) of dry needling of the upper trapezius muscle towards supraspinatus	Numerical Pain Rating Scale (NPRS) but the shoulder range of motion (ROM) and trigger point pressure pain threshold (PPT)	PE seems to be more effective than TDN in relieving pain and improving ROM and PPT supraspinatus values in patients with supraspinatus tendinopathy, both right after treatment and at one-year follow-up.	No adverse events
**B De-la-Cruz-Torres, 2020** [47]	Ultrasound-Guided Percutaneous Needle Electrolysis in Dancers with Chronic Soleus Injury: A Randomized Clinical Trial	RCT	Human	YES	30 dancers randomly allocated to a PE group (n = 10), an eccentric exercise group (n = 10), or a combined group (n = 10)	M: 27; F: 3	21.03 ± 2.88 (16–26)	PT	Chronic soleus injury	/	Eccentric exercise program	/	4 weeks	Two sessions of US-guided PE therapy (one session per week), acupuncture needle measuring 0.30 mm × 40 mm, intensity of 2.5 mA, during 3 s, 3 times (2.5 : 3: 3)	Pain (NRS), ankle dorsiflexion range of motion (DROM) measured using the weight-bearing lunge test (WBLT), endurance, the heel raise test, the Dance Functional Outcome Survey (DFOS) questionnaire, and the minimal clinically important difference (MCID)	US-guided PE, combined with an eccentric exercise program, is a useful therapeutic tool for the treatment of chronic soleus injury	N.A.
**Al-Boloushi Z, 2020** [40]	Comparing two dry needling interventions for plantar heel pain: a randomised controlled trial	RCT	Human	YES	102	M: 30; F: 72	48.8 ± 8.8, (24–60)	PT	Plantar heel pain	NOT	stretching	NOT	4, 8, 12, 26 and 52 weeks	Four sessions of DN or PNE once a week; needle from 30 to 75 mm in length and 0.25 to 0.30 mm in diameter. Intensity of 1.5 mA (intensity was adapted to patients’ characteristics according to their pain tolerance)	Foot Pain domain of the FHSQ, VAS, Quality of life (QoL) was assessed with the EQ-5D-5L	Both PNE and DN were effective for PHP management, reducing mean and maximum pain since the first treatment session, with long-lasting effects (52 weeks) and significant differences between groups in the case of QoL at 52 weeks in favour of the PNE group	No adverse events
**María Pilar López-Royo, 2021** [33]	A Comparative Study of Treatment Interventions for Patellar Tendinopathy: A Randomized Controlled Trial	RCT	Human	YES	48 (19 for each group: control group, DN intervention combined with EE group, or PNE intervention combined with EE)	M: 42; F: 6	18–45, (32.46)	PT	patellar tendinopathy	/	Eccentric exercise	/	up to 22 weeks	An intensity of 3 mA galvanic current was used during the 3 s that the procedure lasted	Disability was measured using the Victorian Institute of Sports Assessment Questionnaire, patellar tendon. VAS, Short Form-36. Ultrasound was used to measure structural abnormalities	DN or PNE combined with an EE program has not shown to be more effective than a program of only EE to improve disability and pain in patients with Patellar tendinopathy in the short (10 wk) and medium (22 wk) terms. Clinical improvements were not associated with structural changes in the tendon.	N.A.
**Fernández D, 2021** [41]	Cost-Effectiveness of Two Dry Needling Interventions for Plantar Heel Pain: A Secondary Analysis of an RCT.	Secondary analysis of RCT	Human	/	102	M: 30; F: 72	48.8 ± 8.8, (24–60)	PT	Plantar heel pain	NOT	YES	NOT	52 Weeks	One session a week of PNE or DN for 4 weeks. Procedure not described in detail	EQ-5D-5L (EuroQoL), the quality-adjusted life years (QALYs)	PNE treatment was more cost-effective than DN, with significant differences at 52 weeks. In the comparisons made according to the cost-effectiveness analysis, this translated into an 86% probability that PNE was more cost-effective compared to DN at 52 weeks	N.A.
**Juan Antonio Valera-Calero, 2021** [37]	Short-term effectiveness of high- and low-intensity percutaneous electrolysis in patients with patellofemoral pain syndrome: A pilot study	RCT	Human	/	15 equally randomized to the high-intensity percutaneous electrolysis (HIPE) experimental group, low-intensity percutaneous electrolysis (LIPE) experimental group or Dry Needling active control group	/	25.6 ± 1.9	PT	Patellofemoral pain syndrome	/	/	/	1 week	The HIPE group received a 660 mA galvanic current for 10 s, the LIPE group 220 mA × 30 s, and the Dry Needling group received no galvanic current; 0.30 × 40 needle	Myofascial trigger points (MTrPs), patellar tendon pain pressure thresholds (PPTs), and subjective anterior knee pain perception (SAKPP)	HIPE and LIPE induce PPT changes in MTrPs and patellar tendon and improvements in SAKPP and seem to produce less pain during the intervention compared with DN	No adverse events
**Sergio Varela-Rodríguez, 2022** [1]	Endogenous Pain Modulation in Response to a Single Session of Percutaneous Electrolysis in Healthy Population: A Double-Blinded Randomized Clinical Trial	RCT	Human	YES	54 asymptomatic subjects randomized into three groups: sham (without electrical current), low-intensity, and high-intensity	M: 34; F: 20	22.96 ± 3.63 (18–40)	PT	asymptomatic	/	/	/	/	low-intensity (0.3 mA, 90 s), and high-intensity (three pulses of 3 mA, 3 s) 0.3 × 25 mm acupuncture needle	Widespread pressure pain thresholds (PPTs), conditioned pain modulation (CPM), and temporal summation (TS) were assessed in the elbow, shoulder, and leg	A single PE intervention modulated pain processing in local and widespread areas, implying an endogenous pain modulation. The pain processing effect was independent of the dosage administrated	No adverse events
**Daniel Fernández-Sanchis, 2022** [36]	A comparative study of treatment interventions for patellar tendinopathy: a secondary cost-effectiveness analysis	Secondary analysis of RCT	Human	YES	48 randomly divided into three groups: PE, dry needling and sham needling	M: 42; F: 6	32.5 ± 7.14	PT	patellar tendinopathy	/	Eccentric exercise: three sets of fifteen repetitions of single-leg squats on a decline board twice a day	/	up to 22 weeks	Percutaneous needle electrolysis (three needle insertions 0.25 mm × 25 mm, intensity of 3 mA, 3 s), dry needling (idem as PE), or sham needling. Four treatment sessions, once every 2 weeks over 8 weeks	Costs, quality-adjusted life years and incremental cost-effectiveness ratio, SF-36 transformed to QoL values using SF-6D scores	The total cost per session was similar in the three groups: EUR 9.46 for the percutaneous needle electrolysis group; EUR 9.44 for the dry needling group; and EUR 8.96 for the sham group. The percutaneous needle electrolysis group presented better cost-effectiveness in terms of quality-adjusted life years and 96% and 93% probability of being cost-effective compared to the sham and dry needling groups, respectively. Percutaneous needle electrolysis has a greater probability of being cost-effective than sham or dry needling treatment	N.A.
**Blanca De-la-Cruz-Torres, 2023** [53]	Ultrasound-Guided Percutaneous Needle Electrolysis Combined with Therapeutic Exercise May Add Benefit in the Management of Soleus Injury in Female Soccer Players: A Pilot Study	Prospective Case–Control	Human	YES	20: an experimental group (exercise program + US-guided PNE; n = 10) or a control group (exercise program + sham stimulation; n = 10)	F: 20	PE: 21.30 ± 3.80 Control: 23.40 ± 5.98	PT	Soleus Injury in Female Soccer Players	/	Specific exercise program (4 sessions per week during 4 weeks) based on strength gluteus exercise, eccentric hamstring exercise, eccentric gastrocnemius exercise, and eccentric soleus exercise	/	4 weeks	PE: needle 0.30 mm × 40 mm, intensity of 1.5 mA, during 3 s, 3 times Control: idem without current	Pain intensity, dorsiflexion range of motion, knee-flexion heel raise test, curve sprint test, and the global rating of change scale	The application of the US-guided PNE combined with a specific exercise program may cause clinical benefits in the treatment of female soccer players with soleus injury	N.A.
**Ana Isabel Benito-de-Pedro, 2023** [51]	Efficacy of Deep Dry Needling versus Percutaneous Electrolysis in Ultrasound-Guided Treatment of Active Myofascial Trigger Points of the Levator Scapulae in Short-Term: A Randomized Controlled Trial	RCT	Human	YES	52—intervention (PE; n = 26) and control (DDN; n = 26) groups	PE: M: 8 F: 19–DDN: M: 8 F: 19	38.77 (36.15–41.38)	PT	non-specific neck pain lasting more than 3 months	/	/	/	14 days	Single treatment session, needle (0.30 mm × 30 or 0.30 mm × 40), 3–5 applications of 5 s at an intensity of 1.5 mA.	Pain intensity: modified visual numeric pain scale (VNPS), pressure pain threshold (PPT), cervical range of motion (CROM), neck disability: Northwick Park Pain Questionnaire (NPQ) and post-needling soreness	PE and DDN appear to have similar short-term effects. PE proved to be a more painful treatment than DDN	No adverse events
**Juan L Sánchez-González, 2023** [44]	Effectiveness of different percutaneous electrolysis protocols in the endogenous modulation of pain: A Double-Blinded Randomized Clinical Trial	RCT	Human	YES	46 (three groups receiving a single ultrasound-guided PE intervention: sham (without electrical current), low-intensity (0.3 mA, 90 s), or high-intensity (three pulses of 3 mA, 3 s) PE	Sham: M: 5; F: 11 Low Int: M: 5; F: 10 High Int: M: 5; F: 10	18–40	PT	asymptomatic	/	/	/	/	A 0.3 × 25 mm acupuncture needle at 45° to the skin in the direction of the lateral epicondyle. The sham group did not receive any electrical current, just the needle insertion; the low-intensity group received an electrical galvanic current at an intensity of 0.3 mA for 90 s, whereas the high-intensity groups received three pulses of 3 mA for 3 s each of electrical galvanic current	Widespread pressure pain thresholds (PPT), conditioned pain modulation (CPM), and temporal summation (TS) were bilaterally assessed in the lateral epicondyle, bicipital groove, transverse process of C5 and the tibialis anterior muscle	One session of PE is able to slightly stimulate modulatory pathways related to nociceptive gain, particularly pressure pain sensitivity and temporal summation	No adverse events
**Jorge Góngora-Rodríguez, 2024** [57]	Structural and Functional Changes in Supraspinatus Tendinopathy through Percutaneous Electrolysis, Percutaneous Peripheral Nerve Stimulation and Eccentric Exercise Combined Therapy: A Single-Blinded Randomized Clinical Trial	RCT	Human	YES	50 randomized in two groups: PE + peripheral nerve stimulation (n = 25) and conventional electrotherapy treatment (TENS + therapeutic US)	M: 36; F: 14	44.24 ± 11.80	PT	Supraspinatus tendinopathy	/	Eccentric exercise program consisting of 3 sets of 10 repetitions of each of the 3 exercises, twice a day, during the 4 weeks	/	up to 24 weeks	Four treatment sessions, one per week, intensity: 350 µA for 72 s, 0.30 × 40 mm acupuncture needles; PNS is carried out after the application of PE, with the same treatment frequency	pain (NPRS), strength, electromyographic activity, ultrasound characteristics of the tendon (echogenicity, thickness, and hypervascularization) and functionality (DASH and SPADI)	Combined treatment with PE, Peripheral Nerve Stimulation, and EE is an effective option, with positive results in the short and long terms	N.A.
**Manuel Rodríguez-Huguet, 2024** [50]	Pulsed negative pressure myofascial vacuum therapy and percutaneous electrolysis in the treatment of lateral epicondylalgia: A single-blind randomized controlled trial	RCT	Human	YES	40	M: 25, F: 15	40.1	PT	Lateral Epicondylalgia	NOT	YES	NOT	1 and 3 months	One weekly session of PNE, for 4 weeks, with a needle 0.3 mm in diameter. Intensity of 3.5 mA for 80 s	Pain, Range of Motion (ROM), pressure pain threshold (PPT), Patient-Rated Tennis Elbow Evaluation Questionnaire (PRTEE)	Pulsed negative pressure myofascial vacuum therapy and ultrasound-guided percutaneous electrolysis, as an adjunct to an eccentric exercise program, is more effective for pain, range of movement, pressure pain threshold, and functionality than manual therapy and ultrasound treatment as an adjunct to the same exercise program in patients with lateral epicondylalgia	No adverse events
**López-Royo MP, 2024** [59]	Functionality and jump performance in patellar tendinopathy with the application of three different treatments	RCT	Human	YES	48 randomized into groups: DN, percutaneous electrolysis (PNE), and sham needling as the control group (CG)	M: 42, F: 6	32.46 ± 7.14	PT	Lateral Epicondylalgia	NOT	Eccentric exercises (three sets of fifteen single-leg squat repetitions on a decline board twice a day)	NOT	Up to 3 months	One treatment session every 2 weeks. Needles (0.25 × 25 mm), for 2 s at 3 mA galvanic current	Spanish version of VISA-P and a jump protocol to assess participants’ performance	Eccentric exercise could be effective in improving functionality in patellar tendinopathy and DN could improve eccentric power in jumps performance. Moreover, the DN group experienced an increase in functionality that correlated with the improvements found in jump performance in eccentric power and concentric strength	N.A.
**Doménech-García V, 2024** [60]	Placebo and nocebo effects of percutaneous needle electrolysis and dry-needling: an intra and inter-treatment sessions analysis of a three-arm randomized double-blinded controlled trial in patients with patellar tendinopathy	Secondary analysis of RCT	Human	YES	48 divided into 3 parallel groups: “no-sham group” (PNE intervention), “single-sham group” (sham PNE by using dry needling), and “double-sham group” (sham PNE by using sham needles).	M: 42, F: 6	35.16 (28–43.5)	PT	Patellar Tendinopathy	NOT	unilateral eccentric exercise program of the quadriceps muscle on the affected side (3 sets of 15 repetitions daily on a decline board)	NOT	/	Every group received four sessions of the needling therapies targeting the patellar tendon over 8 weeks	Clinical pain reduction after needle intervention (placebo) and needle-related pain intensity after needle intervention (nocebo)	Needling therapies for individuals with patellar tendinopathy are prone to elicit placebo effects regarding clinical pain and nocebo effects regarding needling-related pain	No adverse events
**Mustafa Turgut Yildizgoren et al., 2025** [46]	Biochemical reactions and ultrasound insights in percutaneous needle electrolysis therapy	Case Report	Human	YES	1	F	45	/	Lateral epicondylitis	/	/	/	4 weeks post-treatment	US-guided PNE, three sessions, 350 μA, 80 sec each	Pain (VAS), function (QuickDASH), ultrasound visualization of gas formation	VAS decreased from 8/10 to 2/10; QuickDASH improved from 56 to 18; hyperechoic foci (gas) seen on US	No adverse events

N.A.: Not available.

**Table 3:** Papers on the use of PE in animal models.

Authors, Year	Title	Study Type	Human/Not Human	US Imaging	Sample Size	Sex	Age: Mean, (Range)	Operator	Type of Disease	Rehabilitation Pre	Rehabilitation During	Rehabilitation Post	Follow-Up	Setting	Outcomes	Results	Adverse Events
**R Margalef, 2020** [61]	Percutaneous Needle Electrolysis Reverses Neurographic Signs of Nerve Entrapment by Induced Fibrosis in Mice	/	Animal	/	46	/	/	/	Mouse model of sciatic nerve entrapment	/	/	/		1.5 mA for 3 s and 3 repetitions in the immediacy of perineural fibrosis	amplitude (peak-to-peak) of the compound muscle action potential (CMAPs)	sciatic nerve was definitively released from its fibrous entrapment	N.A.
**José Luis Sánchez-Sánchez, 2020** [5]	Changes in Gene Expression Associated with Collagen Regeneration and Remodeling of Extracellular Matrix after Percutaneous Electrolysis on Collagenase-Induced Achilles Tendinopathy in an Experimental Animal Model: A Pilot Study	/	Animal	/	15 divided into three different groups (no treatment vs. percutaneous electrolysis vs. needling)	/	8 weeks	/	Achilles tendon tendinopathy	/	/	/	/	3 sessions (one per week) Each percutaneous electrolysis session consisted of three punctures targeting the Achilles intra-tendon 2 mm away from the osteotendinous junction. The intensity of the continuous (galvanic) electrical current was set at 3 mA and applied for 4 s on each puncture	genes involved in tendon repair and remodeling and histological tissue changes	percutaneous electrolysis increases the expression of some genes associated with collagen regeneration and remodeling of extracellular matrix	N.A.

N.A.: Not available.

**Table 4:** Cadaver studies.

Authors, Year	Title	Human/Not Human	US Imaging	Sample Size	Sex	Age: Mean, (Range)	Operator	Type of Disease	Follow-Up	Setting	Outcomes	Results
**Sergio Borrella-Andrés, 2022** [65]	Application of Percutaneous Needle Electrolysis Does Not Elicit Temperature Changes: An In Vitro Cadaveric Study	Cadaver	YES	10 cryopreserved knees	M: 5; F: 5	67–85	/	/	/	Three applications for 3 s of 3 mA of intensity (3:3:3) when the tendon was the targeted tissue, three applications for 3 s of 1.5 mA of intensity (1.5:3:3) when the fat or muscle was the targeted tissue, and 24 s of 1 mA of intensity (1:24:1) in all tissues	Temperature changes in target tissues after PE application	The application of two different PE protocols did not produce appreciable thermal changes in the tendon, fat, and muscle tissues of human cadavers
**Pedro Belón-Pérez, 2022** [62]	Cadaveric and Ultrasound Validation of Percutaneous Electrolysis Approaches at the Arcade of Frohse: A Potential Treatment for Radial Tunnel Syndrome	Five healthy volunteers (ultrasound study) and three Thiel-embalmed cadaver forearms	YES (Human)	5 humans + 3 cadavers	/	/	PT	Radial tunnel syndrome	/	Human: Two approaches were taken, the first one with the forearm in supination and the second one with the forearm in pronation, both with the elbow straight Cadaver: The needle was inserted into the cadaver with all the tissues overlaid and left in situ during the anatomical dissection to determine if the tip of the needle properly reached the supinator muscle	Ability to reach the target tissue	Accurate needle penetration of the supinator muscle was observed in 100% in both US-imaging and cadaveric studies. No neurovascular bundle of the radial-nerve deep branch was pierced in any insertion
**Laura Calderón-Díez, 2022** [63]	Cadaveric and Ultrasound Validation of Percutaneous Electrolysis Approach at the Achilles Tendon as a Potential Treatment for Achilles Tendinopathy: A Pilot Study	human + cadaver	YES	10 healthy volunteers + 10 fresh cadaver legs	M: 4; F: 6	45 ± 14 years	PT	Achilles tendon tendinopathy	/	A needle was inserted from the medial to the lateral side under the body of the Achilles tendon, just between the tendon and the Kager’s triangle, about 5 cm from the insertion of tendon in the calcaneus Humans: 25 × 0.3 mm filiform solid needle	Ability to reach the target tissue	Percutaneous electrolysis can be safely performed at the Kager’s fat-Achilles tendon interphase if it is US-guided.
**Laura Calderón-Díez, 2025** [64]	The Safety of Ultrasound-Guided Needle Approaches for Patellar Tendinopathy: A Theoretical Cadaveric Model	human + cadaver	YES (Human)	10 healthy humans, 10 knees from 5 cadavers	Humans: M: 7; F: 3 Cadavers: M: 2; F: 3	Humans: mean 42 (males), 33 (females); Cadavers: mean 69 (males), 75 (females)	PT	Patellar tendinopathy	/	Cadaveric dissection (non-US-guided) Ultrasound-guided needling in healthy volunteers	Anatomical safety of needle approach, visualization of infrapatellar nerve branches, safety of lateral approach	No neurovascular bundle of infrapatellar nerve branches was pierced in any insertion from the lateral side; lateral approach considered safe; medial approach vulnerable to nerve injury
**Miguel Malo-Urriés, 2025** [66]	Quantitative Ultrasound Characterization of Intensity-Dependent Changes in Muscle Tissue During Percutaneous Electrolysis	Cadaver	Yes (quantitative ultrasound imaging)	29	M: 50%	73.7 ± 9.94	Not specified	None	None (cadaveric medial gastrocnemius muscle)	/	0.00–10.00 mA. Initial intensity increments were set at 0.10 mA, followed by 0.50 mA increments up to the maximum intensity (10.00 mA). Each application lasted exactly one second	Dose-dependent changes in ultrasound parameters; Muscle_Electrolysis_Dose variable explained 66.7% of dose variance; significant differences between low, medium, and high doses; identified a 1–4 mA therapeutic window for muscle response

**Table 5:** Study of quality assessment using Newcastle–Ottawa scale for case–control studies. Each asterisk represents whether individual criterion within the subsection was fulfilled.

References	Selection				Comparability of Cohorts	Ascertainment of Exposure	Outcomes	Non Response Rate	NOS Score
	Adequate Case Definition	Representativeness of Cases	Selection of Controls	Definition of Controls			Same Method of Ascertainment		
Iborra-Marcos [38]	**	*	*	**	*	*	**	-	10
De-la-Cruz-Torres [53]	**	*	*	**	*	*	*	-	9
García Bermejo [35]	*	*	*	*	*	**	**	-	9

**Table 6:** Study of quality assessment using JBI Critical Appraisal Checklist for Case Reports Studies.

References	Were Patient’s Demographic Characteristics Clearly Described?	Was the Patient’s History Clearly Described and Presented as a Timeline?	Was the Current Clinical Condition of the Patient on Presentation Clearly Described?	Were Diagnostic Tests or Assessment Methods and the Results Clearly Described?	Was the Intervention(s) or Treatment Procedure(s) Clearly Described?	Was the Post-Intervention Clinical Condition Clearly Described?	Were Adverse Events (Harms) or Unanticipated Events Identified and Described?	Does the Case Report Provide Takeaway Lessons?
Abat [31]	Y	-	Y	-	Y	Y	Y	Y
Valera-Garrido [45]	Y	-	Y	-	Y	Y	Y	Y
Abat [32]	Y	Y	Y	-	Y	Y	Y	Y
Moreno [52]	Y	Y	Y	Y	Y	-	Y	-
Jiménez-Rubio [48]	Y	-	-	-	Y	-	-	-
Yildizgoren [46]	Y	Y	Y	Y	Y	Y	Y	Y

**Table 7:** SYRCLE Risk of Bias table for animal studies: 0 = no; 1 = yes.

References	Sequence Generation	Baseline Characteristics	Allocation Concealment	Random Housing	Blinding of Caregivers/Investigators	Random Outcome Assessment	Blinding of Outcome Assessor	Incomplete Outcome Data	Selective Outcome Reporting	Other Sources of Bias
Sánchez-Sánchez [5]	0	1	0	0	0	0	0	1	1	1
Margalef [61]	1	1	0	0	0	0	0	1	1	0

**Table 8:** QUACS Risk of Bias scale: 0 = no; 1 = yes.

References	Clear Aim of the Study	Adequate Sample Description	Inclusion/Exclusion Criteria	Detailed Dissection Procedure	Appropriate Instrumentation	Anatomical Landmarks Described	Intra-Observer Variability Controlled	Inter-Observer Variability Controlled	Outcome Measurement Described	Procedural Reproducibility	Statistical Analysis Performed	Sample Size Justification	Conflict of Interest Disclosed
Borrella-Andrés [65]	1	1	0	1	1	1	0	0	1	1	1	0	1
Belón-Pérez [62]	1	1	1	1	1	1	0	0	1	1	1	0	1
Calderón-Díez [63]	1	1	1	1	1	1	0	0	1	1	1	0	1
Calderón-Díez [64]	1	1	0	1	1	1	0	0	1	1	0	0	1
Malo-Urriés [66]	1	1	0	0	1	1	0	0	1	1	1	0	1

## Data Availability

No new data were created or analyzed in this study. Data sharing is not applicable to this article.
